# Survival as a clinical outcome and its spiritual significance in a cohort of patients with advanced central pelvic neoplastic disease undergoing total pelvic evisceration: a poorly debated issue

**DOI:** 10.3389/fmed.2023.1173687

**Published:** 2023-06-09

**Authors:** Antonio Macciò, Elisabetta Sanna, Roberta Piras, Fabrizio Lavra, Valerio Vallerino, Giovanni Maricosu, Emanuela Giglio, Antonio Mura, Marcello Tidore, Clelia Madeddu

**Affiliations:** ^1^Unit of Obstetrics and Gynecology, Department of Gynecologic Oncology, ARNAS G. Brotzu, Cagliari, Italy; ^2^Department of Surgical Sciences, University of Cagliari, Cagliari, Italy; ^3^Bishopric of Iglesias, Iglesias, Italy; ^4^ASL 8, Cagliari, Italy; ^5^Department of Medical Sciences and Public Health, University of Cagliari, Cagliari, Italy

**Keywords:** spirituality, quality of life, total pelvic evisceration, minimally invasive surgery, survival, spiritual well being, cancer, Gynecologic Oncology

## Abstract

**Background:**

Patients with either treatment-resistant or relapsing advanced central pelvic neoplastic disease present with a condition responsible for debilitating symptoms and consequently poor quality of life (QoL). For these patients, therapeutic strategies are very limited and total pelvic evisceration is the only option for relieving the symptoms and increasing survival. Of note, taking charge of these patients cannot be limited to increasing their lifespan but must also be aimed at improving the clinical, psychological, and spiritual conditions. This study aimed to prospectively evaluate the improvement in survival and QoL, focusing on spiritual wellbeing (SWB), in patients with poor life expectancy who underwent total pelvic evisceration for advanced gynecological cancers at our center.

**Patients and methods:**

The QoL and SWB were assessed using the European Organisation for Research and Treatment of Cancer QoL questionnaire (EORTC QLQ-C30), EORTC QLQ-SWB32, and SWB scale, which were repeatedly administered: 30 days before surgery, 7 days after the procedure, 1 and 3 months after surgery, and then every 3 months until death or the last follow-up assessment. Operative outcomes (blood loss, operative time, hospitalization, and incidence of complications) were evaluated as secondary endpoints. The patients and their families were included in a dedicated psycho-oncological and spiritual support protocol, which was managed by specifically trained and specialized personnel who accompanied them during all phases of the study.

**Results:**

A total of 20 consecutive patients from 2017 to 2022 were included in this study. Of these patients, 7 underwent total pelvic evisceration by laparotomy and 13 underwent laparoscopy. The median survival was 24 months (range: 1–61 months). After a median follow-up of 24 months, 16 (80%) and 10 patients (50%) were alive at 1 year and 2 years after surgery, respectively. The EORTC-QLQ-C30 scores significantly improved yet at 7 days and at 1, 3, 6, and 12 months, as compared with the preoperative values. In particular, an early improvement in pain, overall QoL, and physical and emotional functions was observed. With respect to the SWB, the global SWB item score of the EORTC QLQ-SWB32 questionnaire significantly increased after 1 month and 3 months, as compared with preoperative values (*p* = 0.0153 and *p* = 0.0018, respectively), and remained stable thereafter. The mean SWB scale score was 53.3, with a sense of low overall SWB in 10 patients, a sense of moderate SWB in eight patients, and a sense of high SWB in two patients. The SWB scale score significantly increased after 7 days, 1 month, and 3 months, as compared with the preoperative value (*p* = 0202, *p* = 0.0171, and *p* = 0.0255, respectively), and remained stable thereafter.

**Conclusion:**

Total pelvic evisceration is a valid approach for improving both survival and QoL in selected patients with advanced pelvic neoplasms and poor life expectancy. Our results particularly underline the importance of accompanying the patients and their families during the journey with dedicated psychological and spiritual support protocols.

## 1. Introduction

### 1.1. Background

Pelvic evisceration refers to the en bloc removal of advanced uterine, adnexal, or vaginal cancer or of a central pelvic neoplastic recurrence together with other pelvic organs (rectum, bladder, and vagina) and eventually (if necessary) with adjacent neurovascular and support structures. Described for the first time by Brunschwig (1), pelvic evisceration is mainly considered a palliative method burdened by high mortality and morbidity rates. Over time, owing to improvements in preoperative planning, intensive care, and reconstructive techniques, the perioperative mortality rate has progressively declined from 22%, as described by Brunschwig, to 2–5% in the most recent case series (2–5). The procedure may be defined as “anterior” when the bladder is removed, “posterior” when the rectum is resected, or “total” when both are removed (6). Currently, candidates for this type of surgery are patients with either treatment-resistant or relapsing advanced central pelvic neoplastic disease. These patients present with an advanced local condition responsible for debilitating symptoms and consequently poor quality of life (QoL). For these patients therapeutic strategies are very limited, and extensive surgical resection is the only option, despite being accompanied by a high risk of intra- and postoperative complications.

Pelvic evisceration is an extremely complex operation performed to achieve two possible objectives, namely (1) for a curative purpose in cases when the disease, despite its advanced state, is completely removable and (2) for purely palliative purpose (7). Patients with oncological diseases can be divided into two fundamental categories: those who will heal and those who will not. Intuitively, the clinical and psychological conditions and social impact of these patients are completely different. Therefore, in patients with diseases that are no longer susceptible to healing, specific multidisciplinary evaluations are mandatory. These comprehensive assessments require not only a rigorous evaluation of the extension of the primary or relapsing neoplastic mass but also, above all, an evaluation of a multiplicity of symptoms responsible for the reduction in functional, psychological, and social capacities with consequent impossibility to perform common daily activities (8). Moreover, they need coherent integration with the patients’ families and the social environments. This objective of palliation, which can only be achieved through a complex multidisciplinary specialist approach, must not only combat the symptoms of the disease and therefore prevent and relieve physical suffering but also act to improve the QoL and, in particular, the spiritual issue, with the aim of finalizing the same meaning of survival by reaffirming and protecting the foundations of human dignity (9). Therefore, taking charge of these patients cannot be limited to the sterile and unreasoned idea of simply wanting to increase their lifespan, but must also be aimed at improving the clinical, psychological, and spiritual conditions of the person projected toward the end-of-life path (10).

As stated above, patients with advanced pelvic neoplasia present important symptoms linked to the involvement of various anatomical structures, characterized in most cases by the presence of pain, intestinal and urinary subocclusion/occlusion with hydronephrosis, fistulas, hematuria, and foul-smelling leukorrhea (8). In most cases, the seriousness of the alteration in the QoL that patients present with is proportional to the extent of the neoplastic disease; consequently, the surgical choice of evisceration must be rigorously planned with the identification of the most appropriate techniques and intervention routes. Patients eligible for this procedure must be adequately motivated and supported. Performing a preliminary psychological evaluation and offering valid preoperative counseling are crucial for the success of the procedure (8). Dialogue with the patient and her support network is essential. Expectations in terms of QoL improvement must be clarified, and how they are in balance with the complexity of the procedure and the consequent anatomical and functional changes that this procedure will entail must be properly understood and then accepted. The possibilities of reconstructive surgery must also be presented and analyzed, evaluating together with the patient the advantages and disadvantages of the various techniques and the impact that these will have on her future daily life. Therefore, the goal is to achieve the right balance between life expectancy and QoL. In such context, spirituality and spiritual support are crucial to take care of the patient in his entirety and give him the possibility to find and discover meaning, purpose, and connection, thus increasing the perception of the wellbeing and the significance of the life-time obtained by the surgical and medical approaches.

### 1.2. Aim of the study

The present study aimed to prospectively evaluate the improvement in survival and QoL, with a focus on spiritual wellbeing (SWB) (primary endpoints), and operative outcomes (secondary endpoints), in all consecutive patients with poor life expectancy who underwent total pelvic evisceration for advanced gynecological neoplasia at our center.

## 2. Patients and methods

This study was reported in accordance with the “Enhancing the QUAlity and Transparency Of health Research” guidelines for reporting observational studies (STROBE statement) (11).

The present study was performed by prospectively analyzing all consecutive patients with very advanced-stage gynecologic cancers who underwent total pelvic evisceration for palliative purpose at the Gynecologic Oncology Department of the Regional Referral Center for Cancer Disease Hospital (ARNAS G. Brotzu, Cagliari, Italy) from January 1, 2017, to January 31, 2022. The inclusion criteria were as follows: patients aged >18 years who had chemotherapy-resistant and/or radiotherapy-resistant advanced or recurrent pelvic neoplasia, presenting severe symptoms related to the disease extent and involvement of various anatomical structures not manageable by medical therapies, such as pain, intestinal subocclusion/occlusion, hydronephrosis, fistulas, hematuria, and foul-smelling leukorrhea, who had a poor life expectancy, irrespective of body mass index (BMI) or a history of previous abdominopelvic surgery. The exclusion criteria were as follows: findings suggestive of a high anesthesiologic risk (ASA IV or higher), presence of contraindications to surgery, and a life expectancy of ≤30 days. This study was conducted in accordance with the ethical principles laid down in the Declaration of Helsinki. The study protocol was notified and approved by the Local Institutional Review Board, in accordance with the National Regulatory Agency for observational trials not involving drugs. The patients provided written informed consent for the surgical procedure, participation in the study, use of the collected data, and use of the images. Demographic, anthropometric, and clinical data were extrapolated from medical records and included age, BMI, and the presence of previous abdominopelvic surgery.

The primary endpoints were the improvement in survival and QoL, with a focus on SWB, in treated patients. Operative outcomes (amount of blood loss, duration of the procedure, hospitalization, and incidence of complications) were evaluated as secondary endpoints. Survival was defined as the time elapsed from surgery to death or the date of the last follow-up assessment. The patients’ QoL and SWB were evaluated using the following validated questionnaires: the European Organisation for Research and Treatment of Cancer Quality of Life Questionnaire (EORTC QLQ)-C30 (12), the EORTC QLQ-SWB32 (13), and SWB scale (14). The EORTC QLQ-SWB32 contains a global SWB item (question 32), with a seven-point response/scoring scale (ranging from 0 = “do not know or cannot answer,” or 1 = “very poor” to 7 = “excellent”). For the purposes of this study, the answers to question 32 were considered a measure of global SWB. The questionnaires were repeatedly administered to the patients at 30 days before surgery, 7 days after the procedure, 1 month, and 3 months after surgery, and then every 3 months until death or the last follow-up assessment. The completed questionnaires were collected electronically or manually during the follow-up visits.

**Figure 1:**
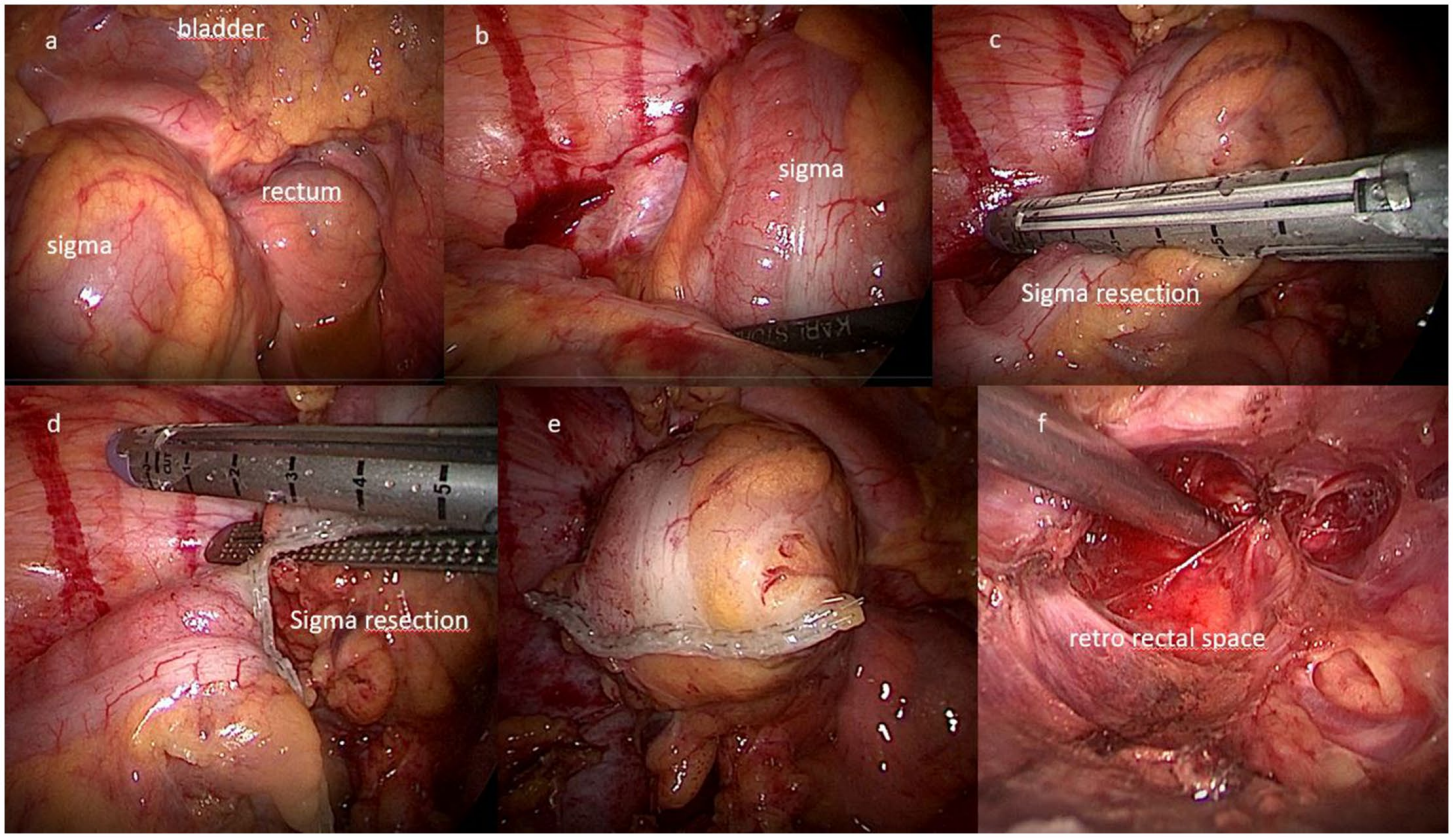
Surgical steps of total pelvic exenteration by laparoscopic approach. **(A,B)** View of the bladder and sigma-rectum; **(C–E)** resection of the sigma by EndoGIA stapler; **(F)** view of the retro-rectal space.

**Figure 2:**
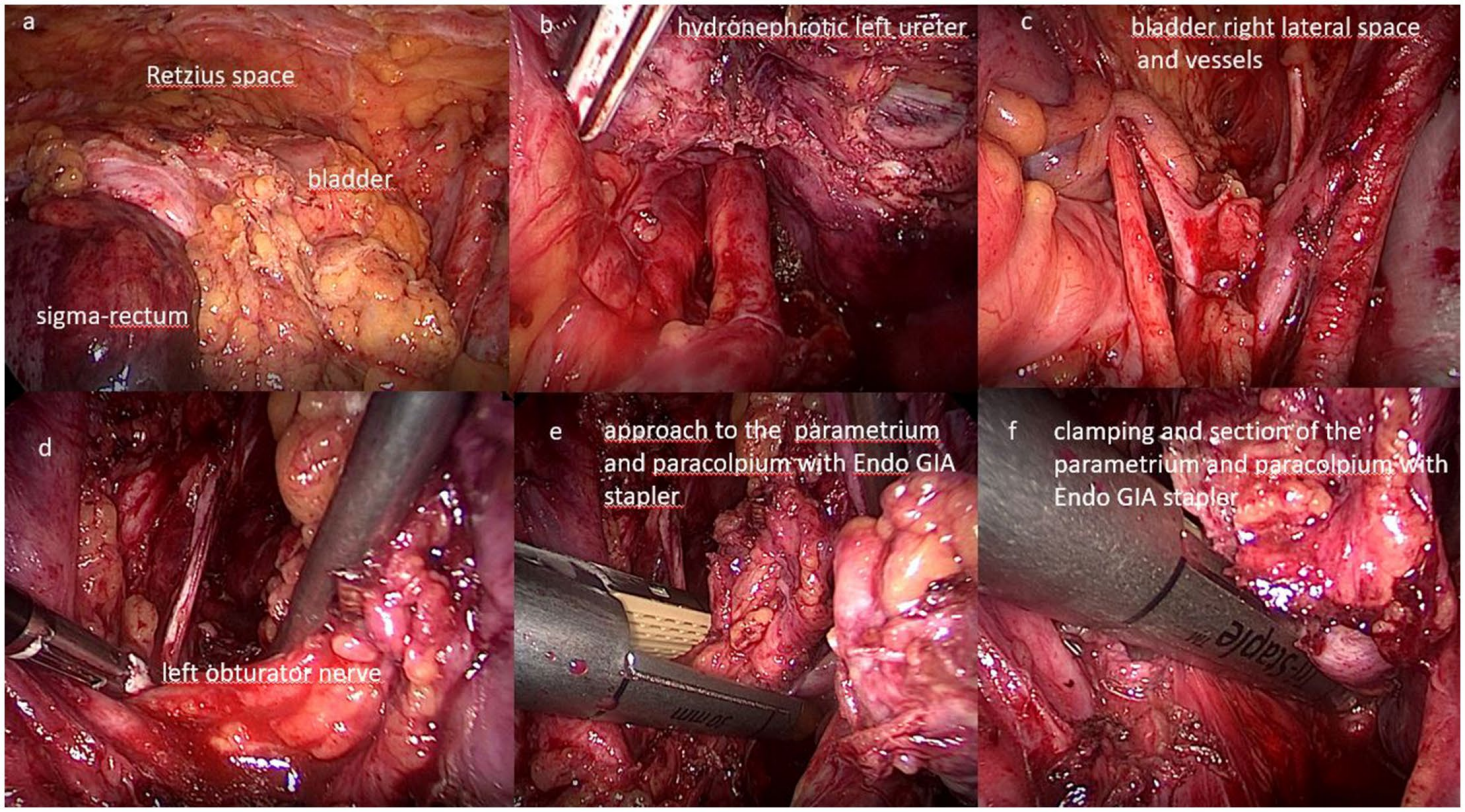
Surgical steps of total pelvic exenteration by laparoscopic approach. **(A)** View of the anterior compartment with the Retzius space and the bladder; **(B)** isolation of the left ureter with hydronephrosis; **(C)** view of the bladder right lateral space and vessels; **(D)** identification of the left obturator nerve; **(E)** approach to the parametrium and paracolpium with EndoGIA stapler; **(F)** clamping and section of the parametrium and paracolpium with EndoGIA stapler.

**Figure 3:**
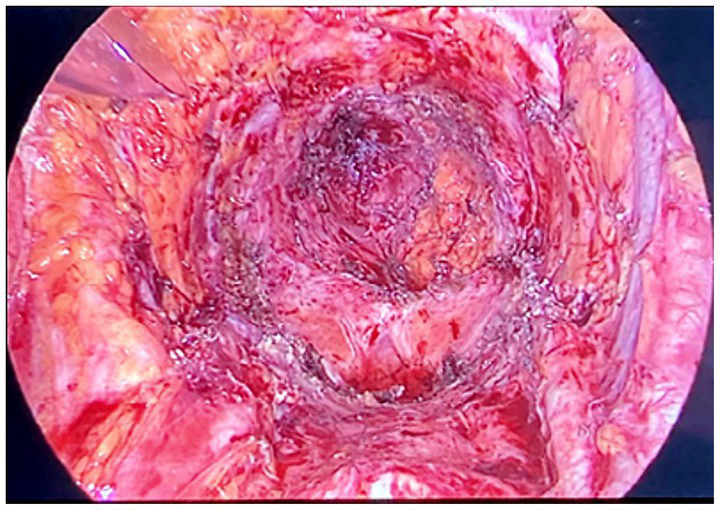
Laparoscopic view of the empty pelvis at the end of the exenterative surgery.

The clinical picture of advanced or relapsing neoplastic disease is associated with an often completely subverted pelvic anatomy, also on account of other previously performed treatments. In these cases, describing and performing a standard procedure is not possible (7), and the operative strategy was modulated based on the highlighted picture and the accessibility of anatomical spaces. An overview and a detailed description of the surgical approach is presented in Supplementary Appendix. Figures 1, 2 show some of the main surgical steps of the laparoscopic surgical approach. Figure 3 presents a laparoscopic view of the empty pelvis at the end of exenteration surgery. All surgical interventions were carefully evaluated, and data on the incidence of intraoperative, postoperative, and long-term complications were collected. Complications were classified and graded according to the Clavien-Dindo classification (15). This classification divides complications into five classes of increasing severity, starting from I, which includes mild complications that do not require specific treatment, to V, which corresponds to the patient’s death. Major complications were defined as grade > III complications. If more than one complication occurred, the complication with the highest degree was included in the analysis. In order to highlight the occurrence of any complications, a daily clinical evaluation was performed postoperatively, and additional tests (e.g., computed tomography with contrast and nuclear magnetic resonance) or exploratory laparoscopy were performed if clinically indicated by the appearance of signs and symptoms (e.g., fever, significant reduction in hemoglobin, bleeding, abdominal pain, bowel obstruction). Routine hematological parameters including leukocyte count (WBC), platelets, lymphocytes, C-reactive protein (CRP), procalcitonin, and fibrinogen were measured. Among these, the blood concentrations of fibrinogen were measured and evaluated daily, and fibrinogen was used as a complication marker to identify any possible inflammation, as described in a work by Macciò et al. (16). In that study, fibrinogen had a higher predictive value for postoperative complications than WBC; in turn, fibrinogen levels were directly related to other inflammation indices such as CRP. After discharge, the patients were instructed to communicate their clinical and health conditions via telephone every 48 h. The patients underwent a clinical evaluation after 1 week and subsequently every 2 weeks (or sooner if symptoms appeared) until progression occurred. Additionally, they were advised to contact the ward if they experienced symptoms attributable to the procedure such as fever, vaginal discharge and/or bleeding, abdominal pain, pelvic discomfort, and constipation. Data regarding possible subsequent hospitalizations, duration of hospitalization, reinterventions, and administered therapies were also analyzed.

**Figure 4:**
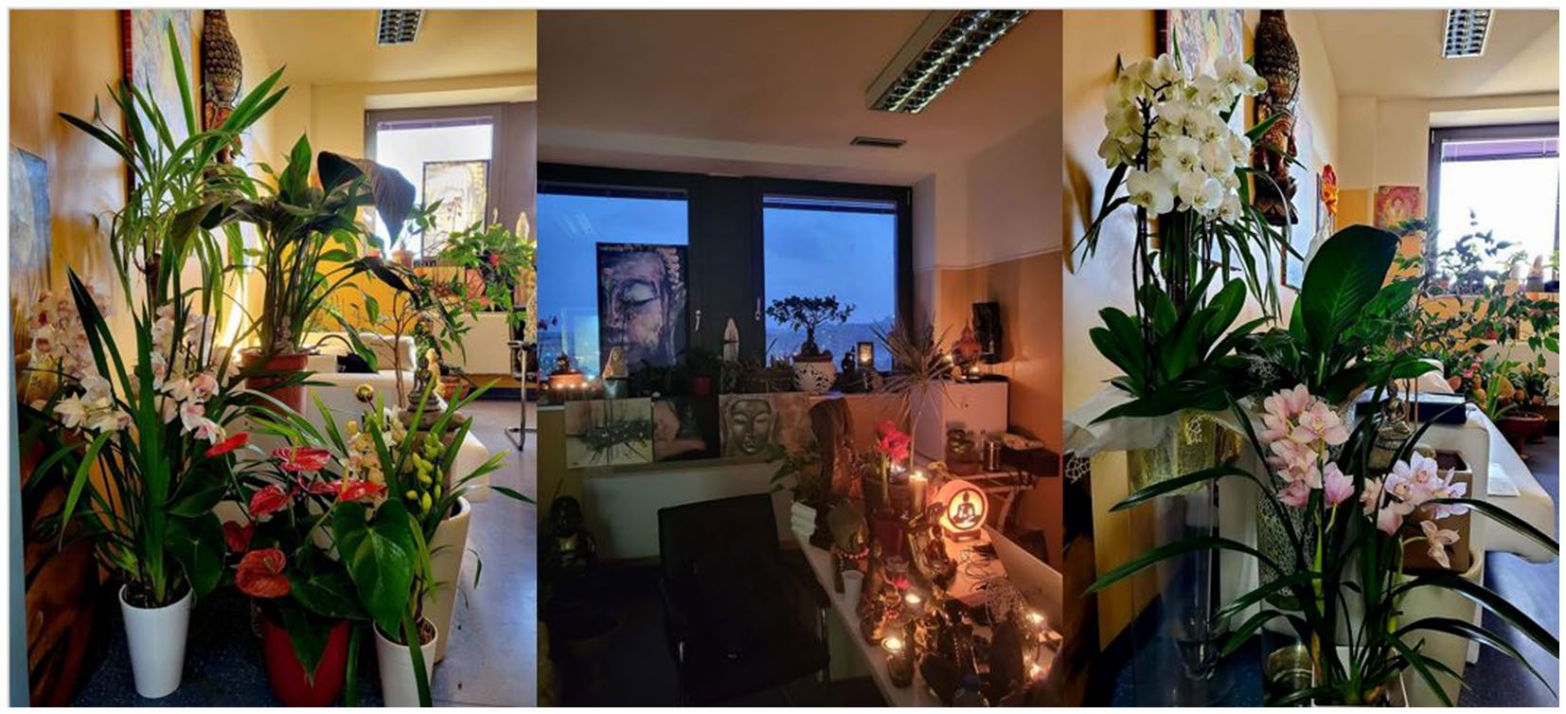
View of the holistic room dedicated to the psychological and spiritual support of the patients enrolled.

The patients and their families were included in a dedicated psycho-oncological and spiritual support protocol, which was managed by specifically trained and specialized personnel who accompanied them during all phases of the study. Psychological and spiritual support was provided in a dedicated and holistic room (Figure 4). Psychological counseling aimed to know the patient’s life history and illness, to evaluate her QoL, to identify needs and any critical issues in terms of emotional reactivity after the communication of the diagnosis of advanced cancer and specifically of the surgical treatment plan. The most common emotional responses are generally alarm and anxiety, coupled with feelings of vulnerability, sadness, hopeless, and fear. However, the reactivity to the diagnosis can also be characterized by disabling emotional experiences such as depression, anxiety, panic and social isolation (17). Therefore, the early identification of critical issues ensures the best possible support to the patient in reorganizing her life in the presence of illness, encouraging the mobilization of resources (personal, family, social) that favor adaptation to treatment and the reduction of psychological discomfort, if experienced, from the time of diagnosis and throughout the course of the disease. In our department, the figure of the hospital psychologist/psychotherapist is an integral part of the multidisciplinary team and guarantees his/her presence in the hospital ward continuously; he/she can also be called by phone as needed. The first consultation was carried out by default with all patients who are candidates for surgery at the time of admission to the ward (or in any case after the doctor has communicated the diagnosis and before surgery). The psychologist/psychotherapist carried out an interview to collect data on the patient’s family, social and work situation and on her QoL, by administering a specific validated questionnaire (i.e., the EORTC QLQ-C30). The different items investigated by the questionnaire may represent a source of psychological distress for the patient, so they must be identified early in order to provide appropriate psychological support during the treatment process. When the distress was moderate/high, continuous support was guaranteed during the course of treatment, which involved the patient and possibly her caregiver/family members. During the psychological counseling the psychologist provided to administer also the SWB questionnaires and take an appropriate spiritual history from the patient. The questions followed the scheme proposed by Puchalski et al. (18). Based on information from the questionnaire and the patient’s spiritual history, the psychologist can identify the presence of a spiritual issue (including spiritual distress or spiritual resources of strength) and make the appropriate referrals to chaplains in the inpatient setting. The chaplain performed the spiritual counseling and helped to identify other spiritual care providers who might be appropriate for the patient in the outpatient setting. Spiritual screenings, histories, and assessments have been documented in patient records (e.g., clinical charts) and made available for use and consultations by all clinicians.

### 2.1. Statistical analysis

Continuous variables were reported as mean ± standard deviation (SD). Categorical data were expressed as absolute number and percentage. Survival was reported as the number of living participants at a given time of follow-up and was represented by a Kaplan–Meier curve. As for QoL and SWB evaluations, the sum of scores obtained by patients for each completed questionnaire was evaluated; changes in scores at different time points versus the baseline were assessed using the Student’s *t*-test for paired data. All reported *p*-values were two-tailed, and *p* < 0.05 was considered statistically significant. All statistical analyses were performed using MedCalc software version 19.6 (MedCalc Software Ltd., Ostend, Belgium).

## 3. Results

**Figure 5:**
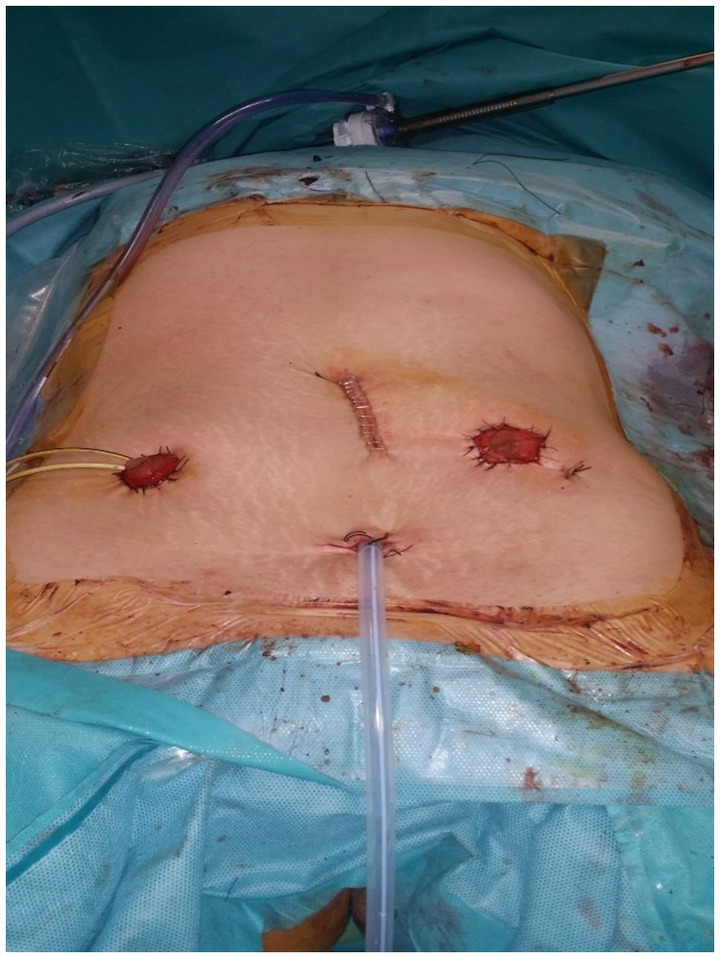
View of the abdomen after the total pelvic evisceration by laparoscopic approach showing the percutaneous left end-colostomy and the right “Bricker” cutaneous uretero-ileostomy.

**Figure 6:**
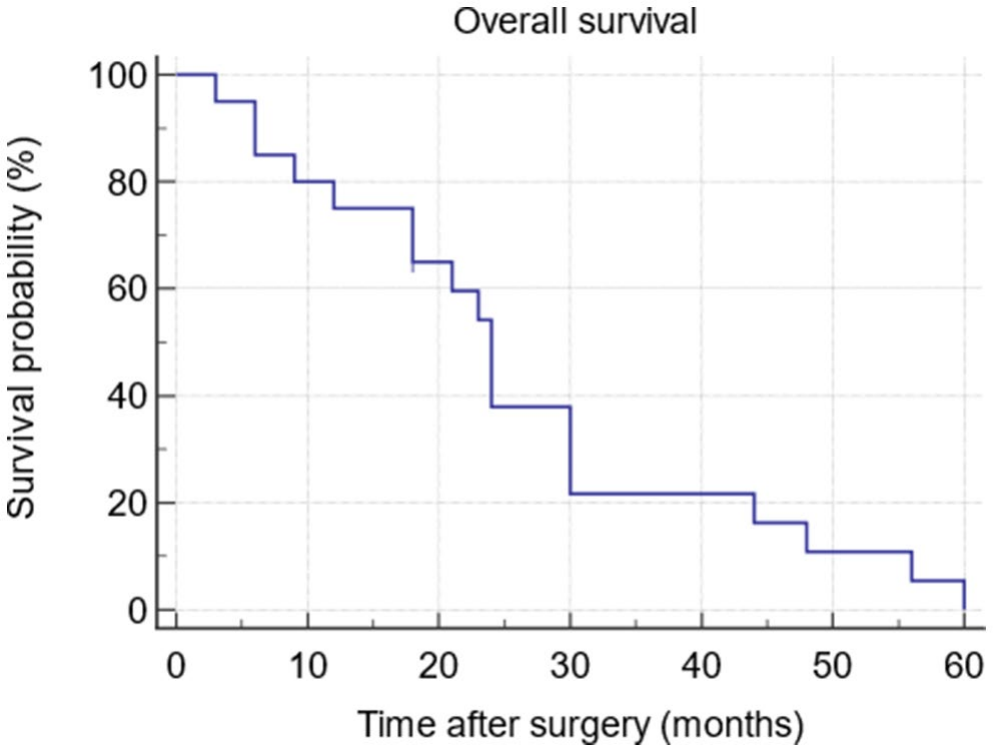
Survival curve by Kaplan-Meyer analysis.

A total of 20 consecutive patients were included in this study; of these patients 7 underwent total pelvic evisceration by laparotomy and 13 underwent laparoscopy. Table 1 summarizes patients’ characteristics and surgical procedures. The mean age was 58.8 ± 4.3 years (range: 40–74 years), and the mean BMI was 22.8 ± 1.7 kg/m^2^ (range: 19–26 kg/m^2^). The indications for surgery were the presence of advanced pelvic cancer in patients with poor life expectancy, with debilitating general and psychological symptoms, and consequently poor QoL. Overall, 14 patients underwent treatment for cervical cancer, 5 patients for endometrial cancer, and 1 patient for clear cell ovarian cancer. Ileal conduit urinary diversion was performed using Bricker’s technique in all patients (Figure 5). Regarding the posterior compartment, among patients for whom the laparotomic approach was used, 4 and 3 patients underwent colorectal anastomosis and colon/sigmoid colostomy, respectively. Among in patients for whom the laparoscopic approach was used, 3 and 10 patients underwent colorectal anastomosis and colon/ sigmoid colostomy, respectively. There were two cases of major postoperative complications (both being grade III according to the Clavien-Dindo classification) in the laparoscopic approach: ureteric leak in one case and colo-rectal anastomosis complication in another case. Additionally, two patients (one for each surgical approach) experienced grade III bowel obstruction due to empty pelvis syndrome within the first 10 days after surgery. No wound hematoma, infection, or late bleeding was observed postoperatively. Table 1 also presents the main postoperative complications. The mean duration of the surgical procedures was 350.5 min (range 300–370 min) for laparotomy and 483.3 min (range 320–700 min) for laparoscopy, with a mean blood loss of 975.7 mL (range 700–1,500 mL) for laparotomy and 300.1 mL (range 100–600 mL) for laparoscopy. The mean length of hospitalization was 15 days (range 8–21 days) after laparotomy and 9 days (range 7–15 days) after laparoscopy (Table 1). The majority of patients quickly recovered, with a relatively rapid return to their daily lives and moderate satisfaction with the anatomical-functional results obtained following the surgical procedure. During the postoperative period, all patients underwent a supportive care protocol specifically developed by our group, which proved to be effective in treating cachexia (19). Figure 6 shows the survival analysis conducted according to the Kaplan–Meier method: the earliest death occurred at approximately 2 months after surgery, the latest at 61 months, with a median survival of 24 months (95% CI: 12–60 months). After a median follow-up of 24 months, 16 patients (80%) were alive at 1 year after surgery, and 10 patients (50%) were alive at 2 years after surgery.

**Table 1:** Patients’ clinical characteristics and operative data.

Patient characteristics	No.	%
Enrolled patients	20	–
Age, years: mean ± SD (range)	58.8 ± 4.3 (40–74)	–
BMI, kg/m^2^: mean ± SD (range)	22.8 ± 1.7 (19–26)	–
Previous abdomino-pelvic surgery	12	60
Primary site of disease		
Cervical cancer	14	70
Endometrial cancer	5	25
Clear cell ovarian cancer	1	5
Previous treatment		
None	–	–
Neoadjuvant CT	2	10
Surgery + (adjuvant) RT ±CT	1	5
Neoadjuvant RT-CT + surgery	16	80
Definitive RT-CT	1	5
Type of exenteration		
Anterior	3	15
Total	17	85
Approach		
Laparotomy	7	35
Laparoscopy	13	65
Urinary diversion		
Bricker’s ileal conduits	20	100
Ureterocutaneostomy	–	–
Neobladder	–	–
Intestinal diversion		
End colostomy	13	65
Colo-rectal anastomosis	7	35
Operative time, min: median (range)		
Laparotomy	350.5 (300–370)	
Laparoscopy	483.3 (320–700)	
Estimated bloss loss, mL: median (range)		
Laparotomy	975.7 (700–1,500)	
Laparoscopy	300.1 (100–600)	
Length of hospitalization, days: median (range)		
Laparotomy	15 (8–21)	–
Laparoscopy	9 (7–15)	–
Major post-operative complications (Clavien-Dindo classification)		
Ureteric leak (grade III)	1	5
Colo-rectal anastomosis complication (grade III)	1	5
Bowel obstruction (grade III)	2	10
Overall Survival, days: median (range)	720 (35–1805)	

BMI, body mass index; CT, chemotherapy; RT, radiotherapy.

### 3.1. QoL and SWB evaluations

Table 2 presents the mean patient-reported scores for the EORTC QLQ-C30 and EORTC QLQ-SWB32 questionnaires and the SWB scale that were collected before surgery, at 7 days after the procedure, and at 1, 3, 6, and 12 months after surgery. Preoperatively, the overall mean QoL score based on the EORTC-QLQ-C30 questionnaire was 38.3 ± 10.4; in the functional scales, the worst scores were reported for physical function, fatigue, overall QoL, and social and emotional functions. Postoperatively, the mean score based on the EORTC-QLQ-C30 questionnaire significantly improved at 7 days, and 1, 3, 6, and 12 months as compared with the preoperative values. In particular, after 7 days and 1 month we observed improvements in symptoms (pain reduction, *p* = 0.0488, 95% CI: −12.811 to −0.0465, and *p* = 0.0230, 95% CI: −30.979 to −3.307, respectively), overall QoL (*p* = 0.0497, 95%CI: 0.0142 to 18.986, and *p* = 0.0145, 95% CI: 0.0142 to 18.986), and emotional function (*p* = 0.0321, 95%CI: 1.032 to 13.768, and *p* = 0.0193, 95% CI: 3.526 to 22.874). Additionally, improvements in overall QoL (*p* = 0.0450, 95% CI: 5.2591 to 43.3123), emotional function (*p* = 0.0017, 95% CI: 13.196 to 28.804), physical function (*p* = 0.0022, 95% CI: 4.9595 to 34.183), fatigue (*p* = 0.0151, 95% CI: −28.227 to −5.373), and pain reduction (p = 0.0022, 95% CI: −34.808 to −12.335) were detected at 2 months. The EORTC-QLQ-C30 score remained stable and significantly higher than the baseline value at the further assessments among survivors. As for SWB, the analysis of the global SWB item score of the EORTC QLQ-SWB32 questionnaire revealed that most patients judged their SWB to be poor, with a mean score of 2.2 ± 1.8. The global SWB item score significantly increased after 1 month and 3 months, as compared with the preoperative values(*p* = 0.0153, 95% CI: 1.232–4.101, and *p* = 0.0018, 95% CI: 2.559–3.707, respectively). It remained stable and significantly higher than the baseline value at the further assessments among survivors. Moreover, the analysis of the SWB scale at baseline indicated a mean score of 53.3, with a sense of low overall SWB (score ranging from 20 to 40) in 10 patients, a sense of moderate SWB (score ranging from 41 to 90) in 10 patients, and a sense of high SWB (score ranging from 91 to 120) in 2 patients. The total score on the SWB scale significantly increased after 7 days, 1 month, and 3 months, as compared with the preoperative value (*p* = 0202, 95% CI: 2.045–8.755, *p* = 0.0171, 95% CI: 10.409–16.390, and *p* = 0.0255, 95% CI: 4.294–24.373, respectively). It remained stable and significantly higher than the baseline value at the further assessments among survivors. Additionally, the number of patients reporting a sense of moderate/high SWB increased from 10 to 14 already at 7 days after surgery.

**Table 2:** Scores of the quality of life and SWB questionnaire before and after surgery.

Questionnaire	Before surgery (No.20) Mean (range)	7 days (No. 20) Mean (range)	*p-*value (95% CI)	1 month (No. 20) Mean (range)	*p*-value (95% CI)	3 months (No. 19) Mean (range)	*p-*value (95% CI)	6 months (No. 17) Mean (range)	*p-*value (95% CI)	12 months (No. 16) Mean (range)	*p-*value (95% CI)
EORTC QLQ-C30	38.3 (20–70)	54 (45–70)	0.0479 (1.232–4.101)	56 (48–70)	0.0345 (6.472–23.806)	61.7 (50–70)	0.030 (0.641–38.692)	80 (65–95)	0.020 (3.526–22.874)	98.3 (90–105)	0.017 (10.641–38.692)
EORTC QLQ-SWB32	2.2 (0–4)	3.3 (1–6)	0.1835 (−2.0743–6.408)	4.67 (3–7)	0.0153 (1.232–4.101)	5.13 (3–7)	0.0018 (2.559–3.707)	5.80 (4–8)	0.0014 (13.196–28.804)	6.12 (4–8)	0.001 (1,965–5.077)
SWB scale	53 (30–85)	57 (35–88)	0.0202 (2.045–8.755)	66.7 (40–99)	0.0171 (10.409–16.390)	74 (40–100)	0.0255 (4.294–24.373)	80 (45–110)	0.0130 (0,841–38.692)	80 (45–120)	0.008 (6,938–14.732)

Data are reported as mean (range). Significance between values was calculated by Student’s *t*-test for paired data. *p* < 0.05 was considered statistically significant. EORTC QLQ, European Organisation for Research and Treatment of Cancer Quality of Life Questionnaire; SWB, spiritual wellbeing; CI, confidence interval. *p-*values was calculated by Student’s *t*-test for paired data versus data before surgery. *P* < 0.05 was considered statistically significant.

## 4. Discussion

### 4.1. Survival as a clinical outcome and its spiritual significance

In the present study, 20 patients with extremely advanced-stage disease, severe impairment of QoL, and poor estimated survival who underwent total pelvic evisceration at a single center because of limited options for further treatment were prospectively analyzed. This study aimed to evaluate the possibility that such a destructive procedure could still achieve an advantage in terms of survival associated with improvements in QoL and SWB. The results confirmed that pelvic evisceration could increase the lifespan in patients with very unfavorable prognosis, for whom it was thought that there were no more possibilities (7, 20, 21). Indeed, we observed a median survival of 24 months (range: 1–61 months), with an overall survival rate of 80% at 1 year and 50% at 2 years after surgery. In this regard, it is not insignificant that in our study, after surgery, the patients received innovative antineoplastic therapies, as all were subjected to molecular characterization and, therefore, to targeted therapy or immunotherapy when an actionable target emerged. Such approach was guided by the fact that the patients included had a chemotherapy-resistant and/or -radiotherapy-resistant advanced or recurrent pelvic neoplasia who progressed with heavily symptomatic disease to standard treatment and for whom a standard chemotherapy was not anymore indicated. Therefore, the good results obtained in terms of survival can be attributable also to the peculiar therapies adopted, thus confirming the clinical significance of survival as evidence of the quality of the chosen treatments. This requires attributing to survival, however, also a spiritual meaning. Indeed, the diagnosis of advanced oncological disease has an impressive overall impact on QoL (physical, psychological, social, and spiritual) derived from the previous clinical history and from the patients’ high perception of imminent death. Therefore, survival certainly has clinical significance, as determined by the therapeutic choices adopted; however, it must be contextualized in terms of the quality of associated life and its undoubted spiritual meaning. In the present study, which intentionally used an emblematic cohort of patients dealing with the above issues, we observed that women complained of very poor QoL during the preoperative phase (in particular, in terms of physical function, pain, fatigue, and social and emotional functions) and state of SWB, as can be highlighted by evaluating the results of the completed questionnaires. After surgery, for all questionnaires, there was a significant improvement, which was already demonstrated in the very early postoperative stages. In detail, we observed an early improvement of pain, overall QoL, physical, and emotional function, as well an early increase of SWB score, yet from 7 days after surgery. Moreover, the improvement remained almost constant over the months until the resumption of the disease, which occurred after a median progression-free survival of 24 months (range: 2–61 months), demonstrating a prolonged beneficial effect of the procedure. The trend of the questionnaires, evaluated from the preoperative phase up to the progression of the disease and then to the final outcome (death or loss of contact with the center), showed an improvement in the physical, psychological, and spiritual condition of our patients. Therefore, we achieved the goal that was set, namely that of dedicating ourselves to the care of patients who could no longer be definitively cured, by directing our work toward giving life with total respect for its quality and spiritual significance.

### 4.2. Definition and centrality of spirituality in cancer patients

Spirituality is generally defined as the set of aspects of experience, not necessarily linked only to the commitment to religious practices but, in general, directed toward the search for a global sense of peace, meaning, purpose, and connection (22, 23). According to a consensus-developed definition, “spirituality is the aspect of humanity that refers to the way individuals seek and express meaning and purpose and the way they experience their connectedness to the moment, to self, to others, to nature, and to the significant or sacred” (18). This concept of spirituality can be found in all cultures and it also involves a search for the ultimate meaning of existence through religion or other paths, including the faith in supernatural being or powers (24). Spirituality includes attitudes, ideals, and values generally related to the spirit or a form of a going-beyond itself radically distinct from matters that nonetheless interact constantly with it (18, 25). Because the spirit is a fundamental intrinsic component of a human being, the spiritual dimension concerns all individuals and constantly accompanies them throughout all phases of their existence (18). During terminal illness, this dimension, together with the questions that it brings, can emerge in a more intense and urgent manner, sometimes in the form of fear, anger, loss, abandonment, conflict, isolation, and bewilderment (26). In any case, a clinician, who tries to give life by attributing a logical clinical meaning to it, must protect its deep spiritual meaning (27, 28). Consistently, care of advanced cancer patients should be centered on spirituality recognizing that, although the patient’s life may be limited, it may be yet full of possibility (18). Cancer can become an occasion to face with fundamental questions of life. Thus, the feeling of existential precariousness triggered by a life-threatening disease as cancer, and by the awareness of the proximity of death can become the origin of a new willingness to live which, although limited in time to live, can be centered on interiority, the strength of the mind, self-care, relationships and feelings, and the hope of “the life to come” (29, 30). Although the deepening, or rediscovery, of their own spiritual dimension in patients with cancer does not necessarily bring the answer to deep existential questions, it can allow the transformation of a serious event into an opportunity for growth and change, even of inner life, which can lead to a more refined, connected, and self-aware existence, emphasizing hope, adaptation, insight and meaning (9). The patient can acquire the awareness that existence is naturally made up of these sudden encounters with pain and suffering and that death is only a phase to go through (31). Many healthcare professionals may consider spirituality, religion, and death taboo topics. The meaning of illness and the possibility of death are often difficult to deal with; however, when one talks about cancer, care, and the body, he/she cannot fail to talk about the spirit (32). Both patients and their caregivers commonly rely on spirituality and religion in search of comfort (33, 34). Therefore, the request for spiritual support is not only a need for a few cancer patients, but also represents an important, sometimes unsatisfied, desire of most of them (35) and, as such, must be guaranteed (36).

### 4.3. Impact of pelvic exenteration surgery on QoL and SWB

The management of women affected by gynecological cancers needs a particular attention even to the preservation of an appropriate QoL, because it can be severely impaired by the different surgical, radiotherapy and chemotherapy treatments received. In this sense, Fagotti et al. (37) evaluated the changes of QoL in a population of patients with advanced ovarian cancer who were randomized to receive primary debulking surgery or neoadjuvant chemotherapy. Of note, they showed that primary debulking surgery, compared with neoadjuvant chemotherapy, was associated with body image deterioration, probably as a consequence of the aggressiveness of surgery and the higher number of ostomies performed. Additionally, although both approaches improved different items of QoL (i.e., physical functioning, emotional functioning, fatigue, pain, dyspnea, insomnia, and appetite loss), the mean score of the different QoL items was better in the neoadjuvant chemotherapy arm than in the primary debulking surgery arm. Conversely, role functioning, cognitive functioning and social functioning improved longitudinally only in patients who were randomized to primary debulking surgery (37).

In the specific setting of patients who were candidates for pelvic evisceration, our approach has proven to be rather peculiar, as most studies in the literature that evaluated the effects of pelvic evisceration on QoL had often focused on the expected QoL reduction resulting from the highly demolitive and reconstructive intervention. Indeed, the impairment in bowel, urinary, and sexual functions related to mutilating surgical approach, such as pelvic exenteration, is considered to have profound psychological implications on women’s QoL, self-identity, and function. At this regard, a retrospective multicenter study by Dessole et al. investigated QoL, emotional distress, and sexuality in patients with gynecological cancer undergoing pelvic exenteration: QoL was measured using the EORTC QLQ-30, QLQ-CX24, and QLQ-OV28 questionnaires administered 12 months after the surgical procedure (38). The analysis of the results revealed that the patients manifested significant psychological discomfort related to disease progression, changes in their physical image, appearance of financial difficulties, gastrointestinal symptoms, and insomnia. The interviewed women also complained of impairments in their body image and physical, role-playing, social, and emotional abilities. The main predictors of poor overall health status and body image were the presence of terminal colostomy, a non-continent urinary bladder, and number of packed ostomies. The authors concluded that long-term psycho-oncological support was strongly recommended and that reducing the number of ostomies was the most effective measure to improve QoL (38). As regard sexuality, Dessole et al. (38) fully evaluated this item only those patients who underwent a vagina-sparing surgery and reported that 29% of them declared to be sexually active, with 60% of them with a good level of sexual enjoyment. At this regard, although the evaluation of sexual dysfunction was not an aim of our study, it should be noted that differently from Dessole et al. (38), the women included in our study represents a specific group of patients with very advanced recurrent and chemo-radiotherapy resistant disease, all candidate to pelvic exenteration with vaginectomy to obtain surgical radicality without possibility of reconstruction due to the advanced stage of disease and high risk of relapse.

In a cohort study on long-term survival and QoL of patients undergoing pelvic evisceration for colorectal cancer, Steffens et al. reported a decrease in QoL indices during the postoperative phase and subsequent improvement at 6 months, with return to preoperative values. The indices then remained almost unchanged in the long term (39). A similar trend was reported by another prospective study conducted by Martinez et al. (40), who assessed the first-year QoL after pelvic exenteration for gynecologic malignancies in 97 patients. They showed a deterioration of QoL and body image during the first 3 months after surgery, with an improvement to baseline level at 1 year after the procedure. In their analysis, elderly patients were the only group category who that constantly reported a reduction in physical and social functions at 1 year after surgery. More recently, Cibula et al. (41) reported acceptable QoL and good therapy satisfaction in a population of 74 patients affected by gynecological cancer who underwent pelvic exenteration and extended pelvic exenteration, without significant difference between the two procedures. The authors’ findings underscored the most reported burdensome aspects related to such surgical approach, mainly in respect to the role and social functions, as well as to negative body image.

Noteworthy, our study highlighted an improvement of the QoL indices from the first phases of the evaluation, showing the positive effects of the procedure on physical wellbeing and psychological and spiritual aspects of the patient after as early as the first week. Notably, the increase in SWB is highly relevant because it is known to be associated with increased hope (42), higher life satisfaction, improved physical and mental health status (28), less depression (43), and less desire for hastened death (44). It should be specified that in our study we did not assess how factors as religion, marital status, palliative care options, and discussion of end-of-life options impacted on SWB response. However, it has been reported that they may influence SWB. In particular, most studies found a positive association between SWB and marital status (i.e., being married or living with a partner) (45–48). Vice versa, the data on the association between religion and SWB are controversial. In particular, a systematic review by Thunè-Boyle et al. (49) found a beneficial effect of religious beliefs and coping in 7 among the 17 included papers, while 3 reported harmful effects and seven no significant effects. More recently, Delgado-Guay et al. (50) did not found any association between religious affiliation and spiritual pain. Concurrent palliative care and discussion of end-of-life options have been associated with improved SWB (51, 52). At this regard, it should be highlighted that in our study each patient after surgery and during the follow up received the appropriate best supportive care for adequate control of physical symptoms as well as a specific multi-agent anticachectic treatment, which may have positively influenced QoL, as previously reported by us (16), as well as SWB.

The results we obtained are probably attributable to the characteristics of the enrolled patients. These patients were those whose seriousness of clinical conditions and the absence of the possibility of recovery determined such a compromise in QoL, such as speculation that the total pelvic evisceration could have brought an improvement. Even our patients experienced profound anatomical-functional alterations resulting from having been subjected to such a destructive operation. However, these alterations were evidently balanced by the results obtained in terms of symptom relief and consequent recovery of dignity, and by the constant psychological and spiritual support. In only one case did we notice a non-prolonged improvement in clinical and psychological/spiritual conditions, which might have been attributed to the patient’s and her spouse’s poor adherence to the proposed psychological and spiritual support protocols.

This point raises the attention on the role of appropriate psychological support in oncologic patients. Evidence from randomized trials demonstrates that psychological supportive interventions may lead both to a survival advantage and to an improvement of QoL in patients with cancer (53, 54). The psychological support is effective mainly in ameliorating the prevalence of specific distressing symptoms, such as emotional distress, anxiety, and depression (54, 55), or in terms of improving adherence to the process of care and survival (56–59). As regard patients with gynecological cancers, a notable study by Arnaboldi et al. (60) aimed to assess the perception and utility of a tailored psychological intervention delivered by a trained psychologists to a population of patients with gynecological cancer candidate to surgery, showed that such approach can promote, as directly reported by patients, their resources to deal with the phase of hospitalization, to face up personal issues in the cancer journey and to face up to returning home after surgery (59, 61). The same group of research in 2015 (62) reported the preliminary results of a non-randomized prospective study on the feasibility of a well-structured psychological management protocol in a population of 49 women with cancer of different sites candidate to a pelvic exenteration procedure. They found that patients experiencing high psychological distress from the start of their clinical pathway and who received intensive psychological support by means of telephone and in-site consultations, showed lower levels of distress from the very beginning of their pelvic exenteration clinical pathway (62).

Notably, as already stated, spirituality is an integrant part of the patient wellbeing, it refers to the meaning and purpose of life and it is essential to give significance to the survival obtained by patients encountering a life-threatening disease as those included in our study. In such context, spirituality assessment and spiritual support add to the psychological counseling the possibility to identify spiritual symptoms, values, and beliefs and to integrate and address them into the plan of care (18). In literature, several authors found that spirituality help both early cancer (63) and advanced cancer patients (50) in coping with their illness, as a source of strength, and showed to have a positive impact on their wellbeing including physical and emotional symptoms (49, 61, 64, 65). Several modalities of spiritual support or psychotherapy interventions integrating spirituality found to improve purpose, meaning, sense of dignity, and will to live, and to lessen sense of suffering (66–71). Of note, some meta-analyses and systematic reviews found that spiritual interventions versus usual psychosocial support obtained a significant positive effect on patient SWB, overall QoL, depression, anxiety, hopelessness, meaning of life (72–74).

### 4.4. Operative outcomes of pelvic exenteration using different surgical approaches

Finally, focusing on the secondary objectives of this work (i.e., evaluation of the operative outcomes), the analysis allowed us to confirm what was reported in the literature. In fact, from the evaluation of the patients’ postoperative courses, we found that laparoscopy was particularly associated with a longer operative time, less intraoperative blood loss, and shorter hospital stay, which is consistent with the previously reported findings of most authors (75, 76). Regarding the incidence of intra- and postoperative complications, our small sample size did not allow us to draw definitive conclusions or to perform a comparison between the two approaches. However, in our series of minimally invasive surgeries, we observed a low incidence of major complications similar to the findings reported recently by some authors (76–78). In particular, a retrospective multicenter analysis carried out by Bizzarri et al. (76) in 23 patients with primary or central recurrent/persistent gynecologic cancer who underwent pelvic exenteration by laparoscopic or robotic approach showed that minimally invasive pelvic exenteration was feasible with low rate of intraoperative and postoperative complications. In their series, early major (grade 2–3) postoperative complications occurred only in 2 patients (8.7%). A literature review performed by the authors (76) showed a low incidence of major intraoperative (only 6.1% for grade 1 complications and 0% for grade 2–4 complications) and postoperative (43.7% for grade 1–2 complications and 12.6% for grade 3–4 complications) complications with minimally invasive pelvic exenteration in comparison to open pelvic exenteration (22–32% for major complications). Other comparative retrospective studies reported a high incidence of overall perioperative complications but did not find any difference between open and minimally invasive pelvic exenteration (79, 80). Another study reported that the two main risk factors for early complication rates (16–71%) seemed to be the extent of tissue damage resulting from radiotherapy and the duration of operation rather than the chosen surgical technique (81). Among the most frequently described complications are the appearance of fistulas between the gastrointestinal system and the skin, urinary tract, or vagina, which we did not observe. Late complications occur in 36–61% of patients and include the appearance of enterocutaneous and vaginal fistulas, ureteral and intestinal occlusion, and pyelonephritis. These would appear to be secondary to the appearance of post-surgical adhesion syndrome, tumor recurrence, and urinary infections derived from repeated self-catheterization (in patients with continent urinary diversion) (82). In particular, Maggioni et al. analyzed the specific operative outcomes by type of surgical approach and highlighted that laparotomic pelvic evisceration was associated with a total morbidity rate of 66%, with 70% of the complications involving the urinary tract and 25% represented by the appearance of intestinal obstruction or fistulas (83). Very recently, Bizzarri et al. (79) published a multi-center, retrospective, observational cohort study on 117 patients undergoing curative and palliative anterior or total pelvic exenteration for gynecological cancer by a minimally invasive approach and an open approach. They reported a low incidence of major complications in both groups, without significant difference in peri-operative morbidity. However, patients treated with minimally invasive pelvic exenteration received fewer intra-operative transfusions. As regard survival outcomes, data obtained by minimally invasive approach resulted comparable those obtained by open approach (78).

Therefore, the minimally invasive approach to pelvic evisceration, as evident in our prospective analysis and also reported by other authors (75, 78) has shown to be feasible and able to reduce the morbidity and consequently improve the patients’ QoL. However, other investigators did not find any differences between minimally invasive and open approach, and the morbidity and perioperative mortality rates remained high (79). Then, the role of minimally invasive surgery for pelvic evisceration needs further evaluation in prospective clinical trials assessing both peri-operative and oncological outcomes (78).

### 4.5. Limitations of the study

The present study has some limitations, mainly its small sample size due to the complexity and specific indication of this peculiar surgical procedure, as well as the single-center recruitment. Moreover, we lacked to evaluate the role of patients’ demographic and social data (as marital status or religious beliefs) and discussion of end-of-life options on SWB response. The strength of this study includes the perspective design and the evaluation of spirituality issues using specifically validated questionnaires, in addition to QoL.

## 5. Conclusion

In conclusion, we can state that total pelvic evisceration, particularly that performed using laparoscopic approach, represents a valid strategy for improving both survival ad QoL in selected patients with advanced pelvic neoplasms and poor life expectancy. The management of such complex pictures requires considerable skill and commitment, careful planning, and the choice of the most appropriate surgical technique. Our experience confirms that, when feasible, the laparoscopic approach is preferred because, in addition to the strictly technical advantages, patients can also benefit from a better aesthetic result and faster recovery. Nonetheless, the adoption of a best supportive therapy alongside with novel targeted therapies after surgery and the use of intensive psychological and spiritual support in our study might have a significantly impact on the results, that cannot be definitely attributed to the surgical procedure alone. In particular, our findings underline the importance of accompanying patients and their families during the journey with dedicated psychological and spiritual support protocols.

Survival is consequent to the best therapeutic choice and therefore has a clear clinical meaning, which, however, must not be separated from spiritual meaning. SWB is a multidimensional construct that is an integral part of the concept of health and incorporates physical and psychological components, as well as existential and “religious” dimensions. The purpose of our study was precisely to combine the common clinical parameters of comparison (type of intervention and complications associated with them) with the need to focus on the protection of total respect for the dignity of the patient in a still little explored area, i.e., that of SWB, which is the primary objective of end-of-life care.

## Data availability statement

The raw data supporting the conclusions of this article will be made available by the authors, without undue reservation.

## Ethics statement

The studies involving human participants were reviewed and approved by the Local Institutional Review Board, Azienda Ospedaliera Brotzu, Cagliari, Italy. The patients/participants provided their written informed consent to participate in this study.

## Author contributions

AMa and CM contributed to the conception and design of the study. AMa, ES, RP, FL, VV, GM, EG, AMu, MT, and CM were involved in the acquisition, analysis and interpretation of the data. MT and CM performed the statistical analysis. AMa, ES, RP, EG, and CM wrote the first draft of the manuscript. All authors contributed to manuscript revision and have read and approved the submitted version.

## Funding

This work was supported by the “Associazione Sarda per la Ricerca in Ginecologia Oncologica” and “Il Giardino di Lu-ONLUS-Insieme per la ricerca sul tumore ovarico.”

## Conflict of interest

The authors declare that the research was conducted in the absence of any commercial or financial relationships that could be construed as a potential conflict of interest.

## Publisher’s note

All claims expressed in this article are solely those of the authors and do not necessarily represent those of their affiliated organizations, or those of the publisher, the editors and the reviewers. Any product that may be evaluated in this article, or claim that may be made by its manufacturer, is not guaranteed or endorsed by the publisher.
