# Supplementary material for: Survival as a clinical outcome and its spiritual significance in a cohort of patients with advanced central pelvic neoplastic disease undergoing total pelvic evisceration: a poorly debated issue
Source: Front Med (Lausanne). 2023 Jun 9;10:1173687. doi: 10.3389/fmed.2023.1173687 (PMC10288149; doi:10.3389/fmed.2023.1173687)
Supplement: Supplementary file 1 [file Presentation_1.PDF]

APPENDIX

A.1. Brief overview of different surgical approaches

When the goal is to improve the patients' QoL, the approach of choice is the one that allows for the achievement of the best result with the least negative impact. The impact of a surgical operation on QoL is strictly dependent on its outcomes, the type of technique used, the possible appearance of perioperative complications (which, in turn, influence the length and quality of hospitalization), and the times of recovery and return to everyday life. In the specific case of total pelvic evisceration, the derivation techniques chosen and their influence on the future daily life of patients deserve specific evaluation [1-3]. With respect to the reduction in blood loss, number of transfusions performed, length of postoperative hospitalization, and complication rate, the data published so far in the literature indicate the superiority of laparoscopy over laparotomy. The laparoscopic approach is associated with a longer operative time; however, this difference decreases as the surgeon's experience increases. In terms of radicality,amount of tissue removed, and recurrence rate, the two methods would appear to be superimposable [4]. Owing to the advent of new technologies, such as automatic staplers, laparoscopy provides surgeons with access to spaces that are otherwise difficult to reach, thereby minimizing tissue damage because of the minor trauma caused, with a positive effect on the postoperative course and the patients' QoL. Consequently, in recent decades, the use of laparoscopy has increased, even though the approach to pelvic evisceration has traditionally been laparotomic since the first case described by Brunschwig. In fact, the use of laparoscopy was initially limited to the preoperative evaluation of patients who were candidates for surgery [5].The first fully laparoscopic pelvic evisceration was described in 2003 by Pomel et al. [6]. Subsequently, other authors described their experiences. One of the largest series was published by Puntambekar et al. [7], who reported the results obtained from 74 patients with cervical cancer treated with laparoscopic anterior evisceration. At the end of the follow-up, all patients, even those in whom recurrence had occurred, reported the absence of local symptoms, demonstrating the validity of evisceration as a palliative treatment. As far as the laparotomic approach for pelvic evisceration is concerned, one of the largest case studies was published by the Maggioni group in 2009 [8]. However, the specific comparison between the two approaches has been the subject of more recent studies [9,10], including a meta-analysis [4]. In detail, the latter analyzed the differences in operative time, blood loss, resection margin status, 30-day morbidity and mortality rates, and length of hospital stay between the two surgical techniques. The authors confirmed what was previously reported in the literature; that is, the operative time for laparoscopy was longer (by approximately 83 min), and minimally invasive surgery was associated with less intraoperative blood loss, lower 30-day morbidity rate, and shorter hospital stay. Thus, when executable, laparoscopy is the approach of choice [4]. However, it is important to emphasize that the patient's QoL was not included among the parameters compared between the two techniques.

A.2. Total pelvic evisceration technique

The clinical picture of advanced or relapsing neoplastic disease is associated with an often completely subverted pelvic anatomy, also on account of other previously performed treatments. Consequently, the surgical strategy was modulated. In these cases, describing and performing a standard procedure are not possible, and the operative strategy is modified based on the highlighted picture and the accessibility of anatomical spaces [11-13]. For explanatory purposes, the surgical steps can be summarized as follows:

Lateral approach:

- Step 1: Definition of the extension of pathology
- Step 2: Opening of the lateral peritoneum (if feasible)
- Step 3: Opening of the lateral avascular, paravesical, and pararectal spaces
- Step 4: Ligation of the uterine/hypogastric artery
- Step 5: Opening of the medial pararectal space.

Anterior approach:
• Step 6: Opening of the anterior peritoneum and vesicouterine and vesicovaginal spaces
• Step 7: Opening of the ureteral tunnel. Cystectomy
• Step 8: Section of the anterior or posterior parametrium and colpotomy
• Step 9: Section of the rectovaginal space until the vulva and perineum
Posterior approach:
• Step 10: Opening of the presacral space. Resection of the sigmoid colon and distal rectum.
Reconstructive phase

The surgical procedure was performed using various devices for hemostasis and dissection, including an ultrasonic dissector (Ultracision® harmonic scalpel, , Ethicon Endosurgery Inc., Cincinnati OH), LigaSure™ Maryland jaw laparoscopic sealer/divider (Covidien, Boulder, CO), LigaSure blunt tip laparoscopic sealer/divider (Covidien, Boulder, CO), BiClamp® LAP and BiClamp® LAP Maryland forceps (Erbe, Germany), 5-mm endo peanut blunt dissector (Covidien, Boulder, CO), 10-mm Endopath™ blunt cherry dissector (Ethicon, Hamburg, Germany), and gauzes. In particular, colic resection and clamping of the parametrium,paracolpium, and associated vessels were performed using the Endo GIA™ stapler (Covidien, Boulder, CO), as shown in Figures 2 and 3. This step represents a useful innovative technique for reducing bleeding and operative time.

A.2.1. Urinary diversion
Urinary diversion refers to the surgical creation of an alternative route to achieve normal urine flow. A derivation can be divided into incontinent, or continent, according to whether the patients can control the flow themselves. An incontinent shunt consists of the entry of the urinary tract into the abdominal skin through a stoma and requires the use of an external collection system. On the other hand, a continent derivation exploits the capacity of another natural sphincter (for example, the anal sphincter) or creates an artificial (internal or external) pocket for urine collection. Incontinent urinary diversion can be temporary (cystostomy, nephrostomy) or permanent; the latter technique mainly involves the execution of “Bricker” cutaneous uretero-ileostomy [14]. The advantage of the latter approach is the lower incidence of infectious complications, which is much more frequent in patients with continent pouches and is related to the impossibility of controlling the urine flow, with consequent discomfort in patients. Therefore, this surgical approach is typically used.

A.2.2. Intestinal derivations
Restoration of intestinal continuity can occur through various ways: colostomy, ileostomy, colorectal or coloanal anastomosis (with or without protective colostomy), and the J-pouch. In this case, the type of derivation is chosen based on the postoperative anatomical picture and the patients’ condition, after in-depth counseling. Both colostomy and ileostomy require the use of an external collection system and have, like incontinent urinary diversion, the disadvantage of the lack of control of fecal flow. The incidence of complications after packing an ostomy varies from 20 to 70% in literature; among these complications, ischemia and necrosis at the ostomy site are affected by the ostomy itself, retraction, parastomal hernia, enterostomal fistulas, stenosis, and skin complications. The incidence of complications is higher in the first postoperative months, prolonging the recovery time and compromising patients’ autonomy [15].

References
1. Husain, A., Curtin, J., Brown, C., Chi, D., Hoskins, W., Poynor, E., et al. (2000). Continent urinary diversion and low-rectal anastomosis in patients undergoing exenterative procedures for recurrent gynecologic malignancies. Gynecol. Oncol. 78(2), 208-211. doi: 10.1006/gyno.2000.5864
2. Angioli, R., Panici, P.B., Mirhashemi, R., Mendez, L., Cantuaria, G., Basile, S., et al. (2003). Continent urinary diversion and low colorectal anastomosis after pelvic exenteration.

Quality of life and complication risk. Crit. Rev. Oncol. Hematol. 48(3), 281-285. doi: 10.1016/s1040-8428(03)00126-4
3. Hawighorst-Knapstein, S., Schönefussrs, G., Hoffmann, S.O., Knapstein, P.G. (1997). Pelvic exenteration: effects of surgery on quality of life and body image--a prospective longitudinal study. Gynecol. Oncol. 66(3), 495-500. doi: 10.1006/gyno.1997.4813
4. PelvEx Collaborative. (2018). Minimally invasive surgery techniques in pelvic exenteration: a systematic and meta-analysis review. Surg. Endosc. 32(12), 4707-4715. doi: 10.1007/s00464-018-6299-5
5. Hatch, K.D. (1998). The role of operative laparoscopy to evaluate candidates for pelvic exenteration. Gynecol. Oncol. 69(2), 93. doi: 10.1006/gyno.1998.5042
6. Pomel, C., Rouzier. R., Pocard. M., Thoury. A., Sideris. L., Morice. P., et al. (2003). Laparoscopic total pelvic exenteration for cervical cancer relapse. Gynecol. Oncol. 91(3), 616-618. doi: 10.1016/j.ygyno.2003.08.032
7. Puntambekar, S., Sharma, V., Jamkar, A.V., Gadkari, Y., Joshi, G., Puntambekar, S., et al. (2016). Our Experience of Laparoscopic Anterior Exenteration in Locally Advanced Cervical Carcinoma. J. Minim. Invasive Gynecol. 23(3), 396-403. doi: 10.1016/j.jmig.2015.12.005
8. Maggioni, A., Roviglione, G., Landoni, F., Zanagnolo, V., Peiretti, M., Colombo, N., et al. (2009). Pelvic exenteration: ten-year experience at the European Institute of Oncology in Milan. Gynecol. Oncol. 2009 114(1), 64-68. doi: 10.1016/j.ygyno.2009.03.029
9. Bizzarri, N., Chiantera, V., Loverro, M., Sozzi, G., Perrone, E., Gueli Alletti, S., et al. (2023). Minimally invasive versus open pelvic exenteration in gynecological malignancies: a propensity-matched survival analysis. Int. J. Gynecol. Cancer. 33(2), 190-197. doi: 10.1136/ijgc-2022-003954
10. Matsuo, K., Matsuzaki, S., Mandelbaum, R.S., Kanao, H., Chang, E.J., Klar, M., et al. (2021). Utilization and perioperative outcome of minimally invasive pelvic exenteration in gynecologic malignancies: A national study in the United States. Gynecol. Oncol. 161(1), 39-45. doi: 10.1016/j.ygyno.2020.12.036
11. Lampe, B., Luengas-Würzinger, V., Weitz, J., Roth, S., Rawert, F., Schuler, E., et al. (2021). Opportunities and Limitations of Pelvic Exenteration Surgery. Cancers. 13(24), 6162. doi: 10.3390/cancers13246162
12. Tejerizo-Garcia, A., Olloqui, A., Lopez, G., Alvarez-Conejo, C., Caso-Maestro, O., Diez A. (2020). Step-by-step total pelviperitonectomy with modified posterior pelvic exenteration. Int. J. Gynecol. Cancer. 30(4), 558. doi: 10.1136/ijgc-2019-000984
13. Macciò, A., Lavra, F., Chiappe, G., Kotsonis, P., Sollai, G., Zamboni, F., Madeddu, C. (2016). Combined laparoscopic excisional surgery for synchronous endometrial and rectal adenocarcinoma in an obese woman. J. Obstet. Gynaecol. 36(8), 1012-1015. doi: 10.1080/01443615.2016.1234444
14. Bricker, E.M. (1950). Bladder substitution after pelvic evisceration. Surg. Clin. North. Am. 30, 1511–1521. doi: 10.1016/s0039-6109(16)33147-4
15. Krishnamurty, D.M., Blatnik, J., Mutch, M. (2017). Stoma Complications. Clin. Colon. Rectal. Surg. 30(3), 193-200. doi: 10.1055/s-0037-1598160
